# Bacterial community of cushion plant *Thylacospermum ceaspitosum* on elevational gradient in the Himalayan cold desert

**DOI:** 10.3389/fmicb.2015.00304

**Published:** 2015-04-16

**Authors:** Klára Řeháková, Alica Chroňáková, Václav Krištůfek, Barbora Kuchtová, Kateřina Čapková, Josef Scharfen, Petr Čapek, Jiří Doležal

**Affiliations:** ^1^Department of Phycology, Ecology, Institute of Botany Academy of Sciences of the Czech Republic, v. v. i.Třeboň, Czech Republic; ^2^Department of Microbial Water Ecology, Biology Centre Academy of Sciences of the Czech Republic, v. v. i - Institute of HydrobiologyCeske Budejovice, Czech Republic; ^3^Department of Soil Microbiology and Soil Chemistry, Biology Centre Academy of Sciences of the Czech Republic, v. v. i - Institute of Soil BiologyČeské Budějovice, Czech Republic; ^4^Department of Botany, Faculty of Science, University of South BohemiaČeské Budějovice, Czech Republic; ^5^Faculty Hospital and Faculty of Medicine, Institute of Clinical Microbiology, Charles UniversityHradec Králové, Czech Republic; ^6^National Reference Laboratory for Pathogenic Actinomycetes, Department of Medical Microbiology and Immunology, Regional Hospital Trutnov, Inc.Trutnov, Czech Republic

**Keywords:** heterotrophic microbial community, subnival soil, life strategy, Ladakh, mountains, Himalayas

## Abstract

Although bacterial assemblages are important components of soils in arid ecosystems, the knowledge about composition, life-strategies, and environmental drivers is still fragmentary, especially in remote high-elevation mountains. We compared the quality and quantity of heterotrophic bacterial assemblages between the rhizosphere of the dominant cushion-forming plant *Thylacospermum ceaspitosum* and its surrounding bulk soil in two mountain ranges (East Karakoram: 4850–5250 m and Little Tibet: 5350–5850 m), in communities from cold steppes to the subnival zone in Ladakh, arid Trans-Himalaya, northwest India. Bacterial communities were characterized by molecular fingerprinting in combination with culture-dependent methods. The effects of environmental factors (elevation, mountain range, and soil physico-chemical parameters) on the bacterial community composition and structure were tested by multivariate redundancy analysis and conditional inference trees. Actinobacteria dominate the cultivable part of community and represent a major bacterial lineage of cold desert soils. The most abundant genera were *Streptomyces*, *Arthrobacter*, and *Paenibacillus*, representing both r- and K-strategists. The soil texture is the most important factor for the community structure and the total bacteria counts. Less abundant and diverse assemblages are found in East Karakoram with coarser soils derived from leucogranite bedrock, while more diverse assemblages in Little Tibet are associated with finer soils derived from easily weathering gneisses. Cushion rhizosphere is in general less diverse than bulk soil, and contains more r-strategists. K-strategists are more associated with the extremes of the gradient, with drought at lowest elevations (4850–5000 m) and frost at the highest elevations (5750–5850 m). The present study illuminates the composition of soil bacterial assemblages in relation to the cushion plant *T. ceaspitosum* in a xeric environment and brings important information about heterotrophic bacteria in Himalayan soil.

## Introduction

Bacteria are a common and fundamental part of microbial communities and constitute a major proportion of biodiversity in soil ecosystems (Gans et al., 2005; Roesch et al., 2007; Fulthorpe et al., 2008; Griffiths et al., 2011). There are two scientific views on their distribution and both opinions have support. The first is that microbial populations can exhibit a geographic distribution (e.g., Fierer and Jackson, 2006; Adler et al., 2007; Griffiths et al., 2011). The second suggests ubiquitous distribution, i.e., everything is everywhere, but the environment selects (e.g., Fenchel and Finlay, 2003; Hubert et al., 2009). The relative importance of contemporary and historical factors in determining spatial patterns in microbial communities can only be evaluated through further studies that systematically sample and record data from various distances, habitats and environmental conditions (Chu et al., 2010).

Bacterial diversity and function are influenced by abiotic parameters, especially pH and nitrogen concentration (Fierer et al., 2009, 2012; Shen et al., 2013), and by biotic interaction with other members of the biotope (Eskelinen et al., 2009; Stark et al., 2012; Ciccazzo et al., 2014). Soil bacteria living in the mountain ecosystems have to withstand harsh environmental conditions such as large temperature and moisture fluctuation and a high intensity of UV radiation (Ley et al., 2004; Zumsteg et al., 2011). Elevational gradients in mountains serve as powerful study systems for answering questions on how the functional diversity and biomass of bacteria can be affected by different microclimatic conditions provided by these gradients. The altitudinal gradient also predicts the composition of the plant community, which will form individual vegetation zones within mountains. The vascular plant species composition directly or indirectly control and/or mediate all multi-trophic interactions in the soil ecosystem (van der Heijden et al., 2008; Chakraborty et al., 2012). To eliminate the influence of plant composition, bacteria in the bulk soil in close vicinity and from rhizosphere of *Thylacospermum caespitosum* were studied. This is the dominant plant of nival and subnival zones of Trans-Himalaya Mountains.

Our hypothesis was that this cushion plant would ameliorate the environmental conditions for microorganisms and would support their survival in subnival zone and consequently influence the species composition of bacteria. This investigation focused on the interaction between this cushion plant and microbial assemblage, and the amelioration effect of *Thylacospermum* on soil bacterial assemblages with emphasis on heterotrophic bacteria.

In this study, two elevational gradients covering the whole distributional range of *T. ceaspitosum* in the Himalaya Mts. were investigated to assess the interactions between bacterial assemblage, soil and microclimatic conditions in different elevations. Composition of bacterial communities was characterized by the method of Capillary Electrophoresis Single Strand Conformation Polymorphism (CE-SSCP) based on 16S rRNA gene (Stach et al., 2001; Zinger et al., 2007, 2008). For more detailed identification of heterotrophic bacterial communities, the culture-depending methods were used.

Our aims were: (1) to evaluate quantitative and qualitative changes in soil bacterial community and physico-chemical properties along an elevational gradient; and (2) to assess the influence of the cushion vascular plant *Thylacospermum ceaspitosum* on the quantity and quality of heterotrophic bacteria and soil bio-chemical characteristics in the nival and subnival zones of Indian Trans-Himalayas.

## Study area

The research was performed in Ladakh, Jammu and Kashmir State, India. The Eastern Karakoram Range delimited study area from the north and the Great Himalaya Range from the south. The region is only rarely affected by monsoonal rains and generally receives very little annual precipitation (<100 mm, Hartmann, 1983; Wang, 1988). Precipitation at lower and middle elevations (3500–5300 m) exceeds evaporation, while above 5300 m the water availability tends to increase due to melting snow from glaciers and occasional spells of rain and snow during summer monsoons (Miehe et al., 2001).

The investigation was done in July and August 2009 at two distinct localities (Tso Moriri, Nubra) where *T. ceaspitosum* is the dominant plant in subnival and nival zones. *Thylacospermum caespitosum* (Caryophyllaceae JUSS., subfamily Alsinoideae (DC.) FENZL, tribus Alsineae LAMARCK and DC.) is one of the most prominent vascular plants in alpine and subnival zones of Ladakh. It is perennial and forms very dense and solid cushions (Klimešová et al., 2011). Its distributional range includes high mountains in Kazakhstan, Kyrgyzstan, NW India, Nepal and China. In the study region, *T. caespitosum* occurs from 4500 to 5900 m elevation (Klimeš and Doležal, 2010).

Tso Moriri is situated in the valley of Lunglung stream on the western side of Chamser Kangri near Tso Moriri Lake (32°59′N, 78°24′E). Tso Moriri geomorphologically belongs to the Tibetan Plateau. Bedrock is formed by siliceous rocks (Precambrian granites, Tso Moriri gneiss) and by calcareous and saline sediments (Phillips, 2008). The cold steppes, alpine grassland and subnival vegetation were characteristic of the studied elevational gradient (Dvorský et al., 2011, 2013). The second locality was a side valley near the village of Tiggur in Nubra Valley, in the northern part of Ladakh (34°45′N, 77°35′E), belonging geomorphologically to the Eastern Karakoram Range. The bedrock mostly consisted of Nubra-Siachen leucogranites (Phillips, 2008). Zonation of the vegetation was similar to the Tso Moriri, but the vegetation zones were shifted downwards by 300–400 m meters because of the higher elevation of surrounding mountains and their large-scale glaciation.

## Materials and methods

### Soil sampling

Soil was collected along two elevational gradients in 2009. In each transect, four sites were selected to cover the entire elevation range of *T. caespitosum*: in Nubra Valley at 4850, 5000, 5100, and 5250 m and in Tso Moriri at 5350, 5600, 5750, and 5850 m. Soil samples were taken from six randomly selected cushions of *T. caespitosum* within each elevation. One soil sample (150 g) was collected from the rhizosphere below the cushion and one sample from the bulk soil outside the cushion. The bulk soil sample was a composite of two samples taken from the eastern and western sides (75 + 75 g). The samples were air dried for 24 h on an aluminum plate, and then placed into sterile 540 ml polypropylene bags (Nasco Whirl-Pak^®^) and transported to the laboratory for analysis. Drying of samples is the only possible soil preservation technique under field conditions in the investigated localities. The closest power supply is accessible after 2 days of traveling and then only irregularly. The soil of Ladakh Mts. is exposed to the drying (average annual precipitation <100 mm) and freezing conditions commonly found in the Himalayas Mts (Bhutiyani et al., 2007). This method of preservation is recommended for arid land soils and BSC samples by Campbell et al. (2010) because it prevents microbial activity in a naturally occurring manner, without the cell damage that may be associated with freezing and, particularly, thawing cycles.

### Measurement of microclimatic conditions

At each elevation site, we recorded air temperature and relative air humidity using a HOBO U23 Pro v2 datalogger placed 10 cm above the soil surface and shielded against direct sunlight. The measurements were recorded every 2 h from August 2008 to August 2011. Additionally, at three sites (Nubra 5000 m, Tso Moriri 5600 m, and 5850 m), we chose one cushion of average size and placed a temperature logger (iButton^®^ DS1923, Maxim Integrated Products) in the soil below it and another one in the soil of the adjacent open area 50 cm from the cushion. Inside the cushions, the loggers were placed 2 cm deep in the substrate under the cushion tissue, where the colonizing species were thought to be rooting. In the open areas, the loggers were buried 2 cm under the soil surface. The measurements were recorded every 2 h from September 2009 to August 2010.

### Physico-chemical characteristics of soil

After transport to the laboratory, the subsamples were oven-dried at 100°C, ground in a mortar and sieved to 2 mm fraction after the removal of roots. The following concentrations of nutrients were determined: total N (TN), P-PO_4_, Ca^2+^, Mg^2+^, Na^+^, and K^+^. Other physico-chemical data were also measured – pH, water content, organic matter content (OM), and texture (percentage content of particles bigger than 0.5 mm in diameter). Soil chemical analyses were conducted in accordance with standardized methods of the Association of German Agricultural Analytical and Research Institutes (VDLUFA 1991). Soil pH was potentiometrically measured in a suspension with 0.01 M CaCl_2_.

### Analyses of bacterial community structure

The pattern of bacterial community structure was obtained by Capillary-Electrophoresis Single Strand Conformation Polymorphism (CE-SSCP), a method which is based on sorting DNA amplicons by electrophoresis under native conditions, according to their length and their nucleotide composition. The acquired SSCP profile is used as a picture of the entire bacterial community (Zinger et al., 2007, 2008).

Soil DNA extractions were carried out in triplicate from 0.25 g of dry soil from each soil sample with the PowerSoil DNA Isolation Kit (MO BIO Laboratories, Ozyme, St. Quentin en Yvelines, France) according to the manufacturer's instructions. DNA extracts from three spatial repetitions of each sample were pooled to make a compounded sample.

The V3 region of bacterial 16S RNA genes was amplified with the primers W49 (5′-ACGGTCCAGACTCCTACGGG-3′) and W104-FAM labeled (5′-TTACCGC GGCTGCTGGCAC-3′) (Delbes et al., 2000). PCR reactions (25 μl) were set up as follows: 2.5 mM of MgCl_2_, 1 U of AmpliTaq GoldTM buffer, 0.4 mg of bovine serum albumin, 0.1 mM of each dNTP, 0.26 mM of each primer, 2 U of AmpliTaqGold DNA polymerase (Applied Biosystems, Courtaboeuf, France), and 10 ng of DNA template. The PCR reaction was carried out as follows: an initial phase at 95°C for 10 min; followed by 30 cycles of denaturation (95°C/30 s), annealing (56°C/15 s), and extension (72°C /15 s) and followed by final step at 72°C for 7 min. The amplicons of each sample were then submitted to CE-SSCP, which were performed on an ABI Prism 3130 XL genetic analyzer (Applied Biosystems, Courtaboeuf, France) (Zinger et al., 2008). The obtained CE-SSCP profiles were normalized and used for statistical analysis.

### Quantification of soil bacteria

Mix-samples were collected from the top 3 cm of the soil at 0.5 m distances from the cushion–bulk soil. Soil below *T. caespitosum* cushions was collected from the boundary line between plant and substrate–rhizosphere soil.

Five grams of field-soil were suspended in 45 ml of 0.2% solution of calgon (sodium hexametaphosphate) and homogenized in an ultrasonic bath (50 kHz, 4 min). Samples were serially diluted (to 10^4^–10^6^) and plated (0.2 ml) in quadruplicate onto R2A agar Difco (pH 7.2) for estimation of cultivable cell population density (colony forming units–CFU) after 8 days of incubation in the dark at 20°C. A colony-forming curve was generated for each soil sample by counting newly visible colonies every 24 h for a 192-h long incubation period and plotting the cumulative number of colonies at each time point (Sigler et al., 2001). Plates that contained between 30 and 300 colonies were selected for enumeration only. “Fast growers” (r-strategists) were defined as bacteria that produce visible colonies within 72 h, “slow growers” (K-strategists) within 73–192 h, respectively. The total number of bacteria (*T*) in each sample was estimated with DAPI (4′, 6-diamidino-2-phenylindole) staining and microscopic counting (Bloem et al., 1995). Cultivable-to-total cell ratio (C/T) (expressed as percentage of CFU from total bacterial counts) was calculated to determine the index of succession state of microbial communities.

### Identification of soil isolates

The isolates for identification using sequencing of 16S rRNA gene were chosen according the growth strategy and character of colony (pigmentation, shape, and consistency). Each strain represented a unique combination of shape, pigmentation, and consistency and speed of growth. Twelve strains of r- and 12 strains of K-strategists from bulk soil were selected. Nine strains of r- and 12 strains of K-strategists from the rhizosphere were chosen. The 16S rRNA gene amplification was performed using universal bacterial primers pA (5′-AGAGTTTGATCCTGGCTCAG-3′) and pH (5′-AAGGAGGTGATCCAGCCGCA-3′) (Edwards et al., 1989), and sequencing. The total volume of PCR reactions was 50 μL. The final reaction mixtures contained (final concentrations) FastStart PCR Master (Roche; 1×) and primers (Metabion; 500 nM each). Cell lysate, prepared by one cycle of freezing and boiling of bacterial culture in water, or genomic DNA (1 μL) served as a template. Thermal cycling was performed as follows: initial denaturation at 94°C for 2 min; followed by 35 cycles of denaturation (94°C/15s), annealing (61°C/30s) and extension (72°C/45s); followed by final extension at 72°C for 5 min. Amplified 16S rRNA genes were purified with the GenElute™ PCR Clean-Up Kit (Sigma-Aldrich) and sequenced using the primers pA and/or pH.

The obtained 16S rRNA gene sequences were edited by Bioedit 7.0.4.1 software (Hall, 1999). The edited sequences were compared against the database of type strains *EzTaxon-e Database* (Kim et al., 2012; http://eztaxon-e.ezbiocloud.net) to retrieve the most relative species. The 16S rRNA gene sequences are available in GenBank under accession numbers KC354443-KC354487.

### Data analyses

In statistical analyses, we distinguish two soil environments: (1) rhizosphere inside cushions and (2) bulk soil from open areas outside cushions.

#### Bacterial patterns derived from CE-SSCP profiles

To test the respective effects of cushion habitat, elevation and soil physico-chemical characteristics and their interactions on the microbial assemblage variation derived from microbial SSCP profiles, we performed a distance-based RDA on a Bray–Curtis dissimilarity matrix. This was calculated from square-root transformed percentage data standardized by sample totals, separately for the two mountain ranges Nubra and Tso Moriri. The variance partitioning procedure in distance-based RDA was performed with explanatory variables and co-variables to remove their effects and to obtain a net effect of an individual predictor. Using this approach, we constructed tests analogous to the testing of particular terms in ANOVA models but for multivariate data; for details, see Lepš and Šmilauer (2003). Differences in bacterial assemblages were tested by 999 permutations. The results of multivariate analyses were visualized in the form of a bi-plot ordination.

To explore further the source of variation in bacterial assemblages in the whole dataset combining Nubra and Tso Moriri, we used multivariate regression trees (MRT) (De'ath, 2002). The MRT hierarchically splits the dissimilarity matrix into the more homogenous subsets according to the selected gradient. We used the same Bray–Curtis dissimilarity matrix as for ordination analyses and pruned the tree according to 1-SE rule (Breiman et al., 1984).

In order to reveal differences in the soil bacteria and physico-chemical parameters between the rhizosphere and bulk soil and their dependence on elevation, we used generalized linear mixed-effect models. The pair-sample identity was a random effect factor, and elevation and soil origin (rhizosphere and bulk soil) were fixed effect factors. The tests were based on the likelihood-ratio approach, approximating the difference in model deviances with a χ^2^ distribution. To control for familywise error rate, the false discovery rate procedure was performed (Benjamini and Hochberg, 1995). Analyses were run using the *lme4* package (R Development Core Team, 2013) in R software.

We further modeled the effect of cushion, elevation, and soil physico-chemical parameters on the soil bacterial variables (total bacteria, C/T, CFU, r-strategy) using conditional inference trees [a type of classification and regression tree (CART)]. This method belongs to non-parametric regressions, making a dichotomous tree which can be used as a predictive model to get some insight into which environmental factors contribute to high/low values of soil bacterial variables. This type of classification and regression tree has several crucial advantages over other approaches (e.g., traditional CART algorithm), including (1) the statistical testing of each split through permutation, (2) no need for problematic pruning of over-fitted trees, and (3) no selection bias toward variables with many possible splits or missing values (Hothorn et al., 2006).

The statistical methods were applied using *Canoco 5* (ter Braak and Smilauer, 2012), *mvpart* (De'ath, 2002), *party* (Hothorn et al., 2006), *lme4* packages within R 3.03 (R Development Core Team, 2013).

## Results

### The response of the bacterial assemblages to environmental factors

**Figure 1:**
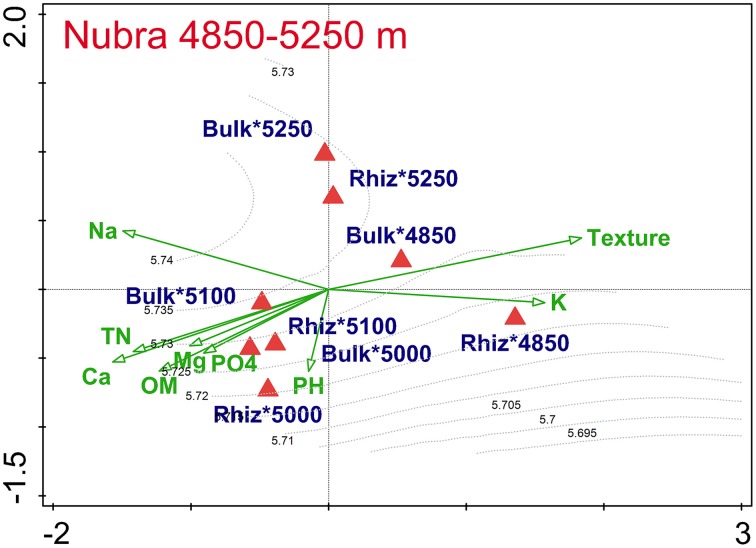
**db-RDA analyses of the bacterial dissimilarity matrices (Bray–Curtis) and vector-fitting of the environmental variables calculated separately for Nubra**. Communities were grouped to the centroid by elevation and cushion interaction.

The combined effect of elevation, cushion habitat, and soil physico-chemical parameters on composition of bacterial assemblages derived from CE-SSCP profiles explained 55.2% of the total data variation in Nubra, which was highly significant (db-RDA, *P* < 0.001). Concerning marginal effects of each explanatory variable (analyses with no covariables), db-RDA in Nubra showed that the contribution to data variation was 38.9% for soil physico-chemical parameters (*F* = 2.1, *P* = 0.008), 30.7% for altitude (*F* = 6.5, *P* = 0.002), and 2.6% for cushion (*F* = 1.636, *P* = 0.088). Variance partitioning revealed that 8.9% was explained solely by elevation, 17.8% by soil physico-chemical parameters, and 0.7% by cushion. Because these three variables were intercorrelated, only the net effect of elevation proved to be significant, while that of soil physico-chemical parameters and cushion became insignificant, being explained by elevation during variance partitioning. This was indicated by the first ordination axis in the ordination diagram that separated less diverse microbial assemblages of the semi-deserts at 4850 m—characterized by coarsest soil, especially outside cushions—from more diverse assemblages at 5000 and 5100 m with higher soil organic matter and nutrient concentrations (Figure 1). The second axis separated the bacterial assemblages of the highest Nubra site at 5250 m from lower elevation sites. Differences in bacterial assemblages between rhizosphere soil and bulk soil outside the cushion were highest at the lowest elevation of 4850 m, and negligible at higher sites in Nubra. Diversity in bacterial assemblages, expressed by the Shannon–Wienner index, increased with elevation and in bulk soil in Nubra, with the lowest values in the rhizosphere soils at 4850 and 5000 m, and highest at the bulk soil at 5250 m.

**Figure 2:**
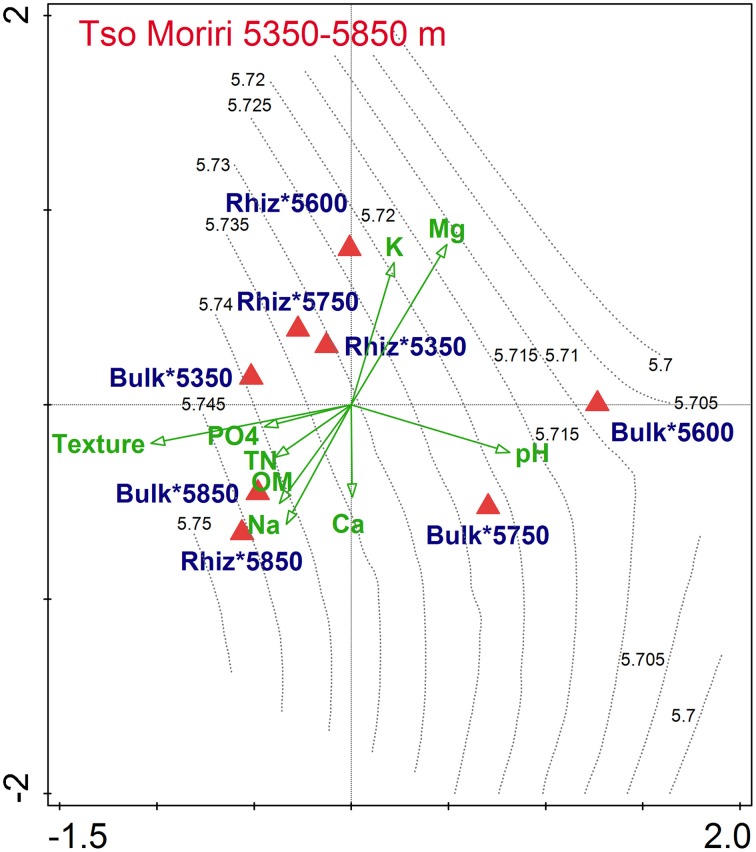
**db-RDA analyses of the bacterial dissimilarity matrices (Bray–Curtis) and vector-fitting of the environmental variables calculated separately for Tso Moriri transects**. Communities were grouped to the centroid by elevation and cushion interaction.

In Tso Moriri, db-RDA showed that the contribution to data variation was non-significant in soil physico-chemical parameters (*F* = 1.1, *P* = 0.46), but was significant for the interaction of elevation and cushion that together explained 24.8% variability in bacterial assemblages (*F* = 6.6, *P* = 0.018). Variance partitioning confirmed the overwhelming effect of elevation, explaining solely 14.8% variation (*F* = 2.5, *P* = 0.048), but also significant was the net effect of the cushion (4.2%, *F* = 1.9, *P* = 0.042), and marginally significant the elevation × cushion interaction (9%, *F* = 1.3, *P* = 0.096). This was indicated by the first axis in the db-RDA ordination diagram that separated less diverse microbial assemblages of the bulk soils at 5600 and 5750 m—characterized by high pH—from more diverse assemblages of bulk soil at 5350 m with higher phosphate nutrient concentrations (Figure 2). The second axis separated the rhizosphere bacterial assemblages at lower elevations—characterized by higher potassium and magnesium—from rhizosphere assemblages at 5850 m with higher OM and concentration of calcium and sodium.

**Figure 3:**
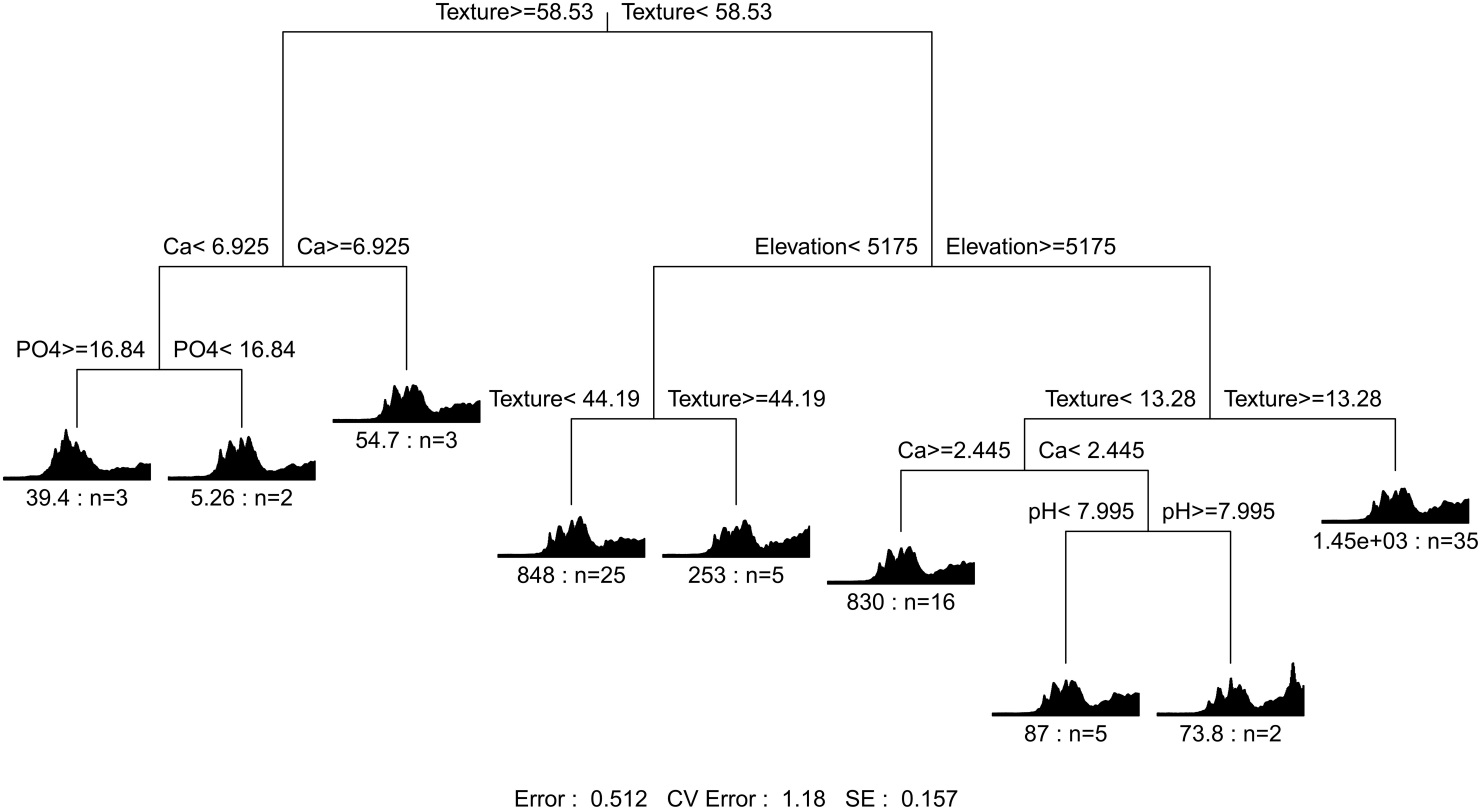
**Environmental factors predicting variation in bacterial assemblages in Nubra and Tso Moriri**. We identified the differences with multivariate regression tree which hierarchically splits bacterial dissimilarity matrix into more homogenous subsets according to the elevation, cushion, and soil physico-chemical parameters. The dissimilarity matrix was calculated from SSCP profiles data with Bray–Curtis index and the tree was pruned according the 1-SE rule.

The multiple regression tree (MRT) analysis (Figure 3) shows that composition of bacterial assemblage variation in the whole dataset (Nubra and Tso Moriri together) was primarily predicted by soil texture (coarseness). Less diverse assemblages from coarser soil on leucogranite bedrock in Nubra were separated from more diverse bacterial assemblages from finer soil at Tso Moriri. The finer soil at Tso Moriri is derived from more easily weathering gneisses. Within coarser soil Nubra assemblages, lower calcium and phosphate concentration separated less diverse rhizosphere communities of lower elevations (<5000 m) from more diverse assemblages of higher elevation sites. Bacterial assemblages in finer texture soils (less than 58.5% of soil particles>0.5 mm) were split in next node by elevation, clearly separating the remaining Nubra sites from all Tso Moriri communities, where texture, calcium and pH play a role in further splitting.

### Growth strategies of cultivable bacteria

**Figure 4:**
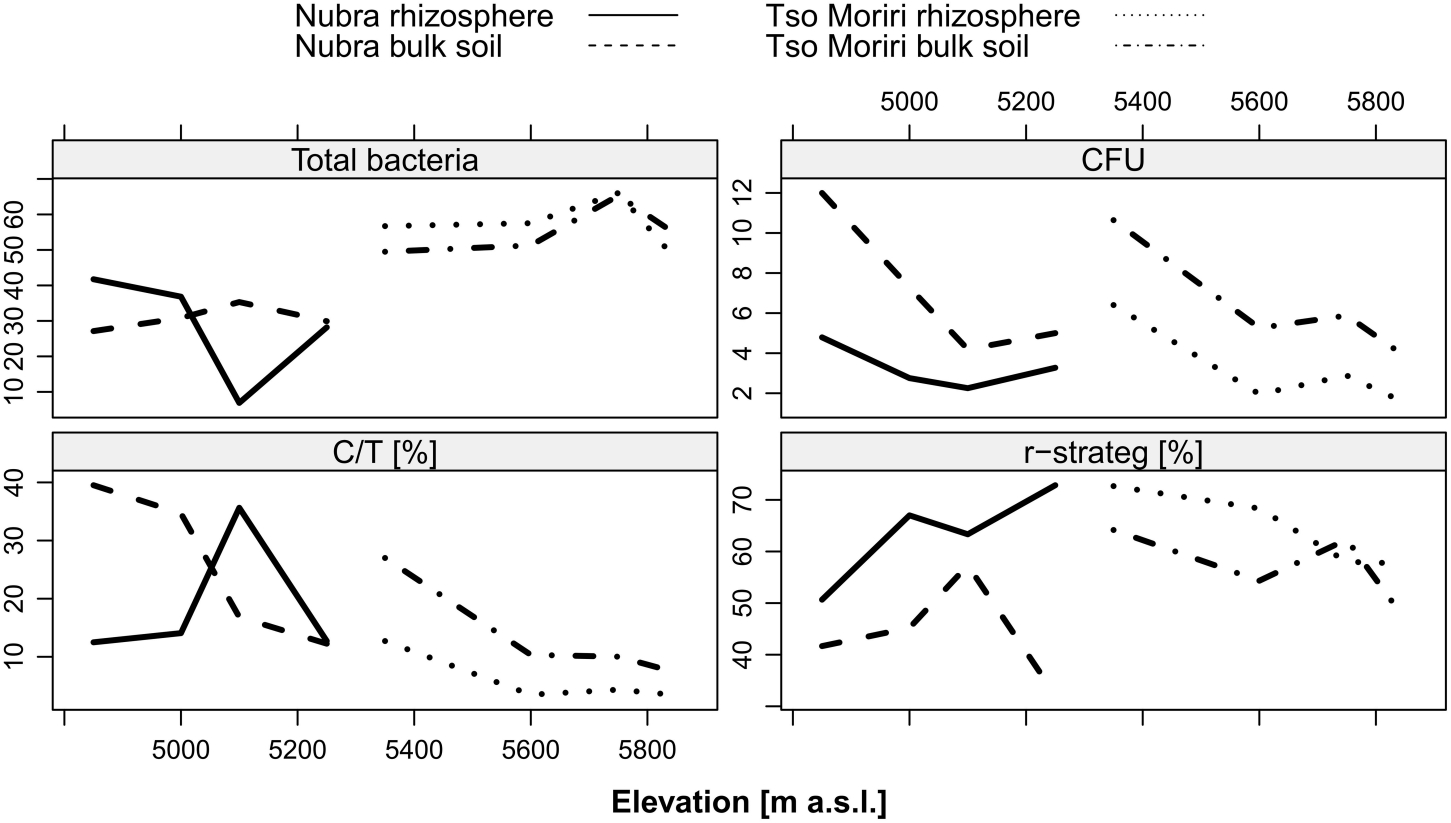
**Characteristics of heterotrophic bacterial communities at two localities Nubra and Tso Moriri in all studied elevations**. Total count of bacteria^*^10^8^/g DW of soil; CFU, culturable cell population density after 8 days of cultivation^*^10^6^/g DW of soil; C/T, percentage of CFU from total bacterial counts; r-strateg—percentage of bacteria that produce visible colonies within 72 h.

An abundant bacterial community was recorded at both localities in all studied elevations (10^8^ cells/g OM). The total bacterial counts were higher in Tso Moriri than Nubra. At both localities, the total bacterial counts were higher in the rhizosphere at the lowest sites (Nubra 4850 m, Tso Moriri 5350 m), whereas more bacteria were recorded in bulk soils at higher sites at Nubra (significant cushion × elevation interaction, Table 1, Figure 4).

**Table 1:** **Microbiological and soil physico-chemical characteristics at two localities Nubra and Tso Moriri in all investigated elevations**.

		**Nubra**						**Tso Moriri**					
		**4850**	**5000**	**5100**	**5250**	**Cushion**	**Elevation**	**5350**	**5600**	**5750**	**5850**	**Cushion**	**Elevation**
Total bacteria 10^8^/g DW	R	**41.7**	36.8	**6.9**	28.2		^**^↓in↑out	56.7	57.5	66.0	47.1		
	B	**27.1**	30.8	**35.3**	29.9			49.5	51.3	65.3	54.3		
CFU 10^6^/g DW	R	**4.8**	**2.8**	2.3	3.3	^***^out>in	^***^↓	**6.4**	**2.0**	**2.9**	**1.5**	^***^out>in	^***^↓
	B	**12.0**	**7.3**	4.2	5.0			**10.6**	**5.3**	**5.9**	**3.8**		
C/T %	R	**12.5**	**14.1**	**35.6**	12.7	^*^out>in	^**^↑in↓out	**12.7**	**3.5**	4.3	3.4	^***^out>in	^***^↓
	B	**39.5**	**34.7**	**16.8**	12.3			**27.0**	**10.3**	10.0	7.3		
r-Strateg %	R	**50.7**	**67**	63.3	**72.8**	^***^in>out	^***^↑in↓out	72.7	68.3	58.0	57.5	^**^in>out	^**^↓
	B	**41.7**	**45.2**	57.3	**32.2**			64.2	54.3	62.3	46.8		
TN mg/kg	R	487	1686	1163	985			833	697	1252	1415		^**^↑
	B	613	1385	778	1024			738	957	1148	1554		
P-PO^3−^_4_mg/kg	R	17.6	19.1	12.6	12.3	^*^out>in	^***^↓	28.2	14.1	24.8	12.7	^*^out>in	^*^↓
	B	**23.0**	**25.7**	14.4	13.0			**39.5**	**20.8**	23.1	17.2		
Ca mg/g	R	5.2	32.6	12.7	17.7			2.9	2.5	2.4	2.5	^*^out>in	^**^↓in↑out
	B	7.3	33.3	17.3	17.6			2.5	3.0	**3.0**	**3.6**		
Mg mg/g	R	8.1	8.5	7.6	7.5	^*^out>in		3.5	2.8	2.5	2.2		^***^↓
	B	7.6	**9.9**	**8.6**	8.3			3.1	3.1	2.6	2.5		
K mg/g	R	6.5	3.7	3.9	3.7		^***^↓	3.0	1.8	2.2	1.9		^*^↓> in
	B	5.9	4.4	4.0	4.1		^***^↑	2.4	2.0	2.2	2.2		
Na mg/g	R	0.6	0.6	0.9	0.9			0.3	0.2	0.5	0.5		^***^↑> out
	B	0.4	0.7	0.9	0.9			0.1	0.2	0.6	0.6		
OM %	R	2.4	**7.2**	**4.4**	2.8	^**^in>out		2.3	2.0	2.6	2.8		
	B	1.8	3.0	2.7	2.9			2.5	2.2	2.3	2.8		
Soil particles>0.5 mm %	R	54.0	19.9	14.4	42.5		^*^↓	11.9	10.6	15.1	20.1		^**^↑
	B	55.4	25.9	18.0	35.9			13.3	10.2	13.4	18.6		
pH	R	8.4	8.5	8.6	8.4	^**^out>in		7.7	7.7	7.1	6.9	^***^out>in	^**^↓
	B	8.5	8.8	**9.0**	**8.8**			**8.1**	**8.3**	**8.0**	**7.8**		

Availability of soil nutrients and composition of microbial assemblages in the rhizosphere (R) T. caespitosum or in the bulk soil (B). An upward or downward pointing arrow indicates a positive or negative relationship between the dependent variable and elevation, based on the likelihood-ratio test in generalized linear mixed-effect models (

*** *P < 0.001*,

** *P < 0.01*,

* P < 0.05).

*Also shown are post-hoc Tukey tests on paired differences (significantly higher values inside/outside cushion are in bold). The presented values are mean from six replications at one elevation*.

The cultivable bacteria isolated from the soils allowed determination of growth strategies. The amount of cultivable bacteria (CFU) was two orders lower (10^6^ cells/g OM) than the total bacterial count (Figure 4). The CFU of aerobic heterotrophic bacterial population represented 1–56% of the total bacterial counts. At both localities, CFU values decreased with increasing elevation and a larger proportion of CFU (2–56%) was found in bulk soil (Figure 4). The lower elevation sites (4850 and 5000 m) in Nubra had significantly higher proportions of CFU in bulk soil (30–56%) outside cushions than inside them, while the higher-elevation sites (5100 and 5250 m) did not differ significantly (Table 1). All elevation sites in Tso Moriri had a higher proportion of CFU outside than inside cushions.

At both localities, the percentage of cultivable bacteria, expressed as culturable-to-total cell ratio (C/T), was higher in bulk soils, with the one exception at 5100 m elevation in Nubra. C/T ratio values tend to decrease with increasing elevation both in the bulk soil and the rhizosphere in Tso Moriri, while in Nubra it tends to increase in the rhizosphere with elevation (significant cushion × elevation interaction, Table 1, Figure 4).

The analysis of life strategy (r/K) of bacteria, based on the time interval needed to form a colony on an agar plate, showed significantly more r-strategists in the rhizosphere than in the bulk soil outside the cushion (Figure 4, Table 1). The significant interaction between cushion and elevation was found in Nubra; the percentage of r-strategists increased in the rhizosphere with increasing elevation, while the opposite was found for the bulk soil outside the cushions. A decreasing trend was observed for both soil environments in Tso Moriri (Figure 4, Table 1).

To reveal the diversity of the cultivable part of bacterial communities, sequencing of 16S rRNA gene was provided on the isolated strains, both for r/K-strategists. The taxonomic composition of r- and K- strategists was different in the bulk soil and the rhizosphere (Table 2). The r-strategists were less diverse than the K-strategists on species, genus, and family and even phylum level. Only four families of r-strategists were determined in both types of studied soils, while seven families from bulk soil and 10 families from rhizosphere were recorded for K-strategists (Table 2). The families of r-strategists found in bulk soil and rhizosphere were the same. Surprisingly, both r- and K- strategists were covered by the phylum Actinobacteria and specialized K-strategists were mostly represented by Firmicutes, Proteobacteria and Cytophaga-Bacteriodes-Flavobacterium groups. The families of K-strategists were more diversified between soil types; only three of them were identical (Micrococcaceae, Streptomycetaceae, and Microbacteriaceae). The other bacteria representing the K-strategists were isolated rarely and also represented the unique bacterial isolates for both types of soils.

**Table 2:** **Species composition and growth strategy of bacteria in bulk soil and rhizosphere of *Thylacospermum***.

	**Bulk soil**		**Rhizosphere**		**Total**	
	**r-strateg**	**K-strateg**	**r-strateg**	**K-strateg**	**r-strateg**	**K-strateg**
**ACTINOBACTERIA**						
**Brevibacteriaceae**						
Brevibacterium luteolum^*^ (94.9%)		**+**				**+**
**Intersporangiaceae**						
*Knoellia locipacati* (98.2%)				**+**		**+**
**Microbacteriaceae**						
*Agrococcus citreus* (99.6%)		**+**				**+**
*Clavibacter michiganensis* (99.4–99.5%)	**+**	**+**			**+**	**+**
*Curtobacterium flaccumfaciens* (100%)			**+**		**+**	
*Microbacterium phyllosphaerae* (98.7%)			**+**		**+**	
*Mycetocola manganoxydans* (99.6%)				**+**		**+**
**Micrococcaceae**						
*Arthrobacter agilis* (99.6%)		**+**				**+**
*Arthrobacter humicola* (99.6%)	**+**				**+**	
*Arthrobacter nitroguajacolicus* (99.7%)	**+**				**+**	
*Arthrobacter oryzea* (98.6%)		**+**				**+**
*Arthrobacter pascens* (99.7–99.9%)	**+**		**+**		**+**	
*Arthrobacter polychromogenes* (99.7%)	**+**				**+**	
*Arthrobacter tumbae* (99.7%)		**+**		**+**		**+**
*Kocuria rosea* (99.5%)	**+**				**+**	
**Micromonosporaceae**						
*Micromonospora saelicesensis* (98.7%)				**+**		**+**
**Nocardioidaceae**						
*Kribbella catacumbae* (99.5%)		**+**				**+**
**Promicromnonosporaceae**						
Isoptericola dokdonensis^*^ (95.2%)				**+**		**+**
**Streptomycetaceae**						
*Streptomyces badius* (99.3%)			**+**		**+**	
*Streptomyces cirratus* (99.7–99.9)	**+**	**+**	**+**		**+**	**+**
*Streptomyces cyaneofuscatus* (100%)	**+**				**+**	
*Streptomyces glomeroaurantiacus* (99.6%)	**+**				**+**	
*Streptomyces humidus* (99.7%)			**+**		**+**	
*Streptomyces litmocidini* (98.3–98.6%)			**+**		**+**	
*Streptomyces niveus* (99.4–99.7%)		**+**				**+**
*Streptomyces scabrisporus* (99.8%)				**+**		**+**
**FIRMICUTES**						
**Paenibacillaceae**						
*Paenibacillus alginolyticus* (98.7%)				**+**		**+**
*Paenibacillus amyloliticus* (99.7%)	**+**		**+**		**+**	
*Paenibacillus borealis* (99.2%)				**+**		**+**
**Staphylococcaceae**						
*Staphylococcus caprae* (100%)				**+**		**+**
*Staphylococcus warneri* (100%)				**+**		**+**
**PROTEOBACTERIA**						
**Alpha-proteobacteria**						
**Acetobacteraceae**						
*Roseomonas aestuarii* (98.6%)				**+**		**+**
**Rhodospirillaceae**						
*Skermanella aerolata* (99.6%)		**+**				**+**
**Beta-proteobacteria**						
**Alcaligenaceae**						
*Pigmentiphaga litoralis* (98.6%)				**+**		**+**
**BACTEROIDETES-CYTOPHAGA-FIRMUCUTES group**						
**Cytophagaceae**						
*Dyadobacter psychrophilus* (98.6%)		**+**				**+**
Total no. species	9	11	8	12	15	22
Total no. genus	5	8	5	10	7	16
Total no. families	4	7	4	10	4	14

*Numbers in parentheses are nucleotide similarity of single strains*.

* *means low nucleotide similarity on genus level*.

Species composition of cultivable bacteria showed small similarity between r/K-strategists and also between the soil types (Table 2). There were 35 species (19 genera) identified at both soil types, with 13 (seven genera) unique in bulk soil, 21 (10 genera) unique in the rhizosphere, and only the *Atrhrobacter, Streptomyces*, and *Paenibacillus* genera were common to both soils. On the species level, only *Streptomyces cirratus* and *Paenibacilus amyloliticus* were identified in both soils, otherwise the species composition did not overlap (Table 2). Two unidentifiable isolates were found in studied soil, and they are potentially species of bacteria new to science (Table 2).

### Cushion, elevation, and soil parameters impact on cultivable bacteria

**Figure 5:**
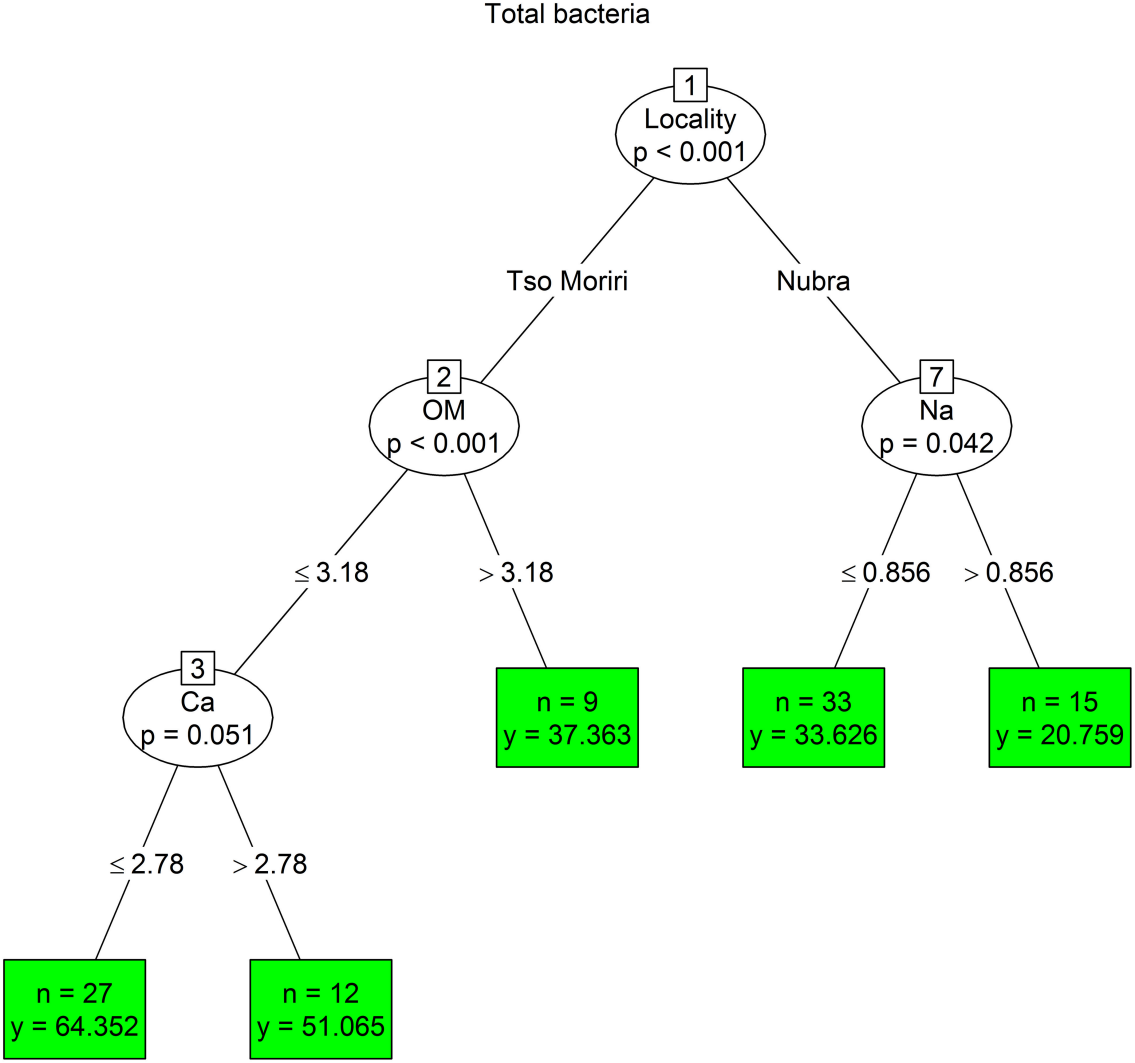
**Conditional inference trees testing the effect of cushion, elevation, and soil physico-chemical parameters on the total bacterial counts**. Hierarchical splitting of the data was based on selecting predictors that best distinguish a variable's responses and dividing samples into two groups according to the predictor's splitting value. Significant predictors are shown in oval windows, while the number of releves in each group (*n*) and their mean species richness (*y*) in green boxes.

The conditional inference tree analysis of total bacteria counts showed primarily a locality effect, separating high-elevation sites of Tso Moriri (5350–5850 m) with more bacteria from Nubra sites (4850–5250 m). Nubra sites were separated at the next node by Na, with significantly more bacteria found in soils with lower concentration of sodium (<0.856 mg/g). At Tso Moriri, more total bacteria was found in soil with lower organic matter (<3.18%) and Ca (<2.78 mg/g) contents (Figure 5).

**Figure 6:**
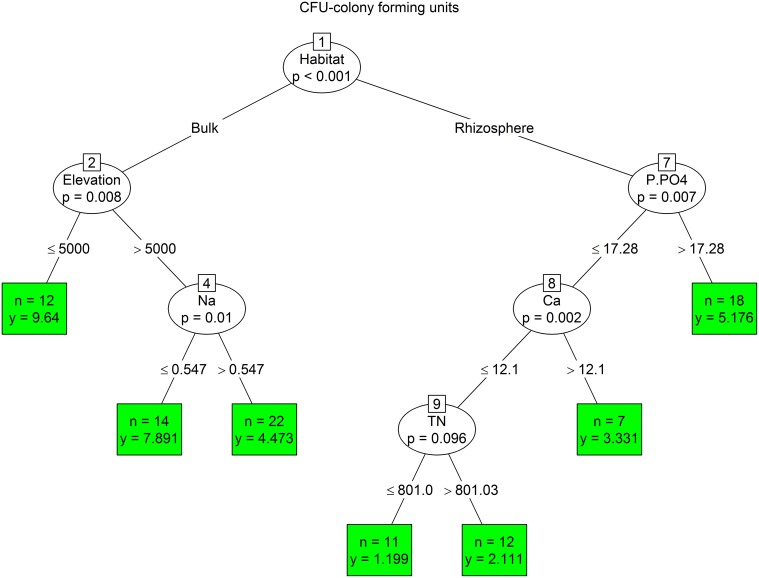
**The conditional inference tree analysis of CFU (for details see Figure 4)**.

The analysis for CFU showed a primary habitat effect, separating bulk soils with higher CFU values from rhizosphere soils. Rhizosphere bacterial communities were separated at the next node by the concentration of phosphate. The higher amount of CFU was presented in less phosphate-limited soils (>17.28 mg/kg). The least CFU in rhizosphere were found in samples with lower concentrations of PO_4_-P, Ca, and total N. The amount of CFU in bulk soils reached a higher number at elevations sites lower than 5000 m, and in soils containing less sodium (Figure 6).

**Figure 7:**
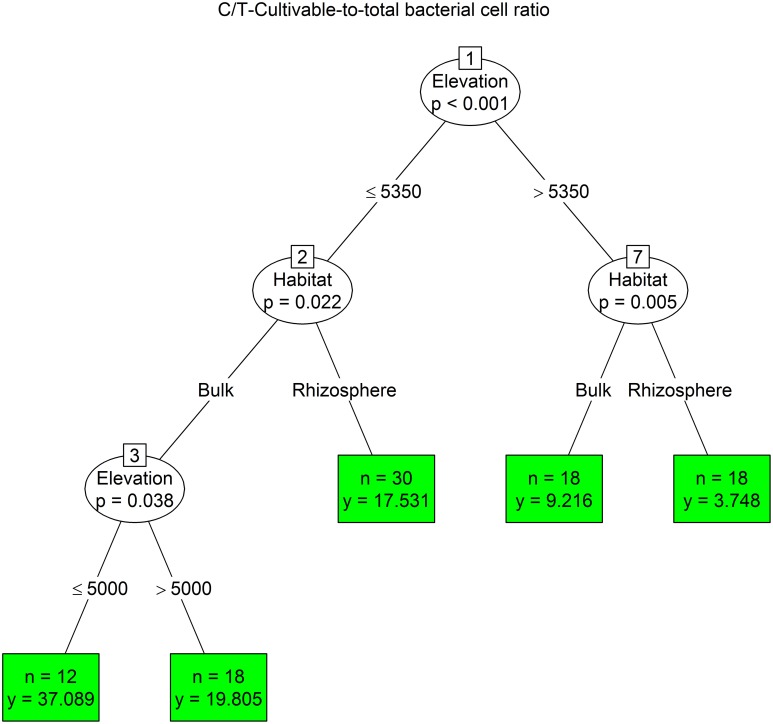
**The conditional inference tree analysis of proportion of cultivable bacteria to total bacterial count (for details see Figure 4)**.

The conditional inference tree analysis of C/T ratio showed primarily an elevation effect, separating lower-elevation sites below 5350 m with higher C/T ratio from the higher-elevation sites (above 5350 m). The next divisions were in both cases based on cushion habitat, with the highest C/T ratio values found in bulk soils from lower elevations below 5000 m (Figure 7).

**Figure 8:**
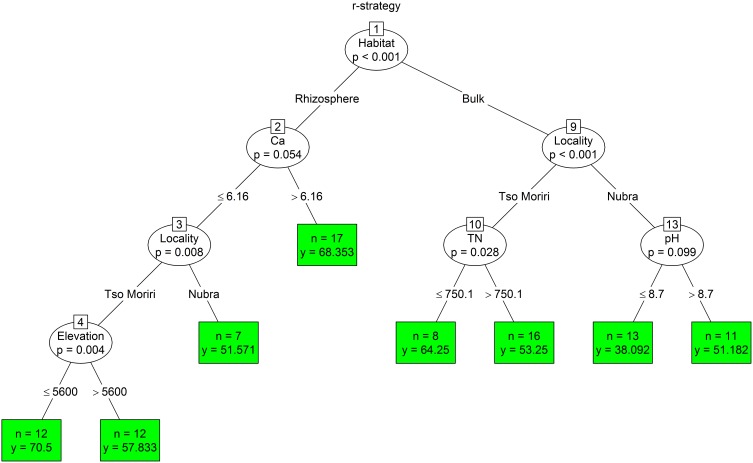
**The conditional inference tree analysis of r-strategist (for details see Figure 4)**.

The analysis for proportion of r-strategists showed a primary cushion habitat effect, separating bulk soils from the rhizosphere. The r-strategists from the rhizosphere were later separated by calcium content, where the critical concentration was 6.16 mg/g. Tso Moriri lower elevation sites (5350–5600 m) had more r-strategists than sites above 5600 m. The abundance of r-strategists in bulk soil was determined firstly by locality. pH influenced the abundance of r-strategists at Nubra sites, where the more abundant communities were found in soil with pH higher than 8. At Tso Moriri the abundance of r-strategists was influenced by TN concentration (Figure 8).

### Microclimatic conditions

**Figure 9:**
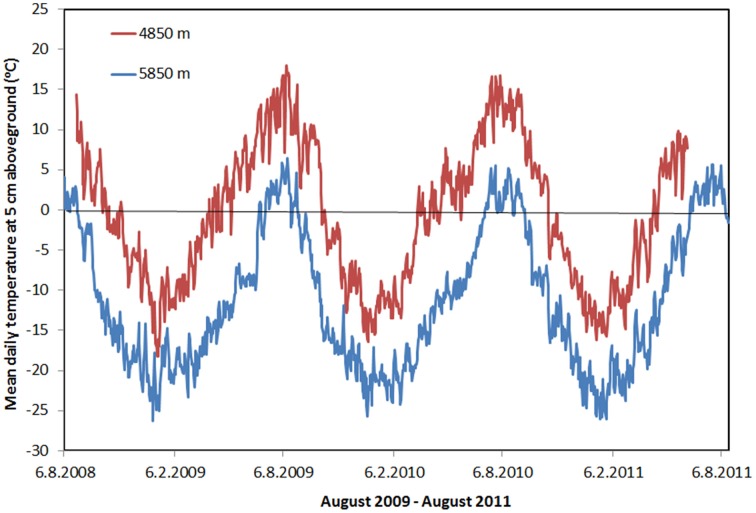
**The course of the mean daily temperature at 5 cm aboveground at the lowest studied elevation (Nubra 4850 m) and the highest (Tso Moriri 5850 m) during years 2008–11**.

At Tso Moriri, the vegetation season, defined as the period with mean daily soil temperatures above freezing lasted nearly 5 months at 5350 m, 3.5 months at 5600 m, and 3 months at 5750 m (data not shown). At the highest elevation (5850 m), the vegetation season was restricted to less than 2 months (56 days, Figure 9). The mean air annual/summer temperatures decreased from −4.4/7.3 to −10.4/4.4°C between 5350 and 5850 m, while relative air humidity increased from 61/50 to 84/53%. The sites differed mainly in the duration of the sub-zero temperature spells over the course of a 24-h period. While air temperature 10 cm above ground never dropped below zero at the lowest elevation during August, it usually fell below zero for about 2–3 h at the middle elevations; at the highest elevation at 5850 m freezing lasted between 6 and 10 h every day, particularly in the second half of August (Figure 9) when many plants still flowered and fruited. Daily air temperatures rose to 8–17°C at all four elevations but for a much shorter time per day at higher elevations. In Nubra, the vegetation season lasted between 3 months (mid-May to beginning of September) at 5250 m and 5 months (the beginning of May to mid-October) at 4850 m (Figure 9). The mean air annual/summer temperatures decreased from −1.6/7.7°C to −3.6/7.1 and the relative air humidity increased from 39/38 to 87/69% with increasing elevation. At all sites, air temperatures in the warmest month of August remained above zero both day and night.

Cushions from a low site in Nubra (5000 m) provided warmer microsites, as measured 2 cm below ground, compared with open areas (annual mean *T* = −0.3 vs. −3.4°C). They also had twice as many degree-days (1227 vs. 601 at *T*_base_ = 0°C) and a frost-free period lasting a month longer (data not shown). On the other hand, the differences at the highest site in Tso Moriri were only minor, the open areas being even slightly warmer than cushions.

### Soil chemical properties

**Figure 10:**
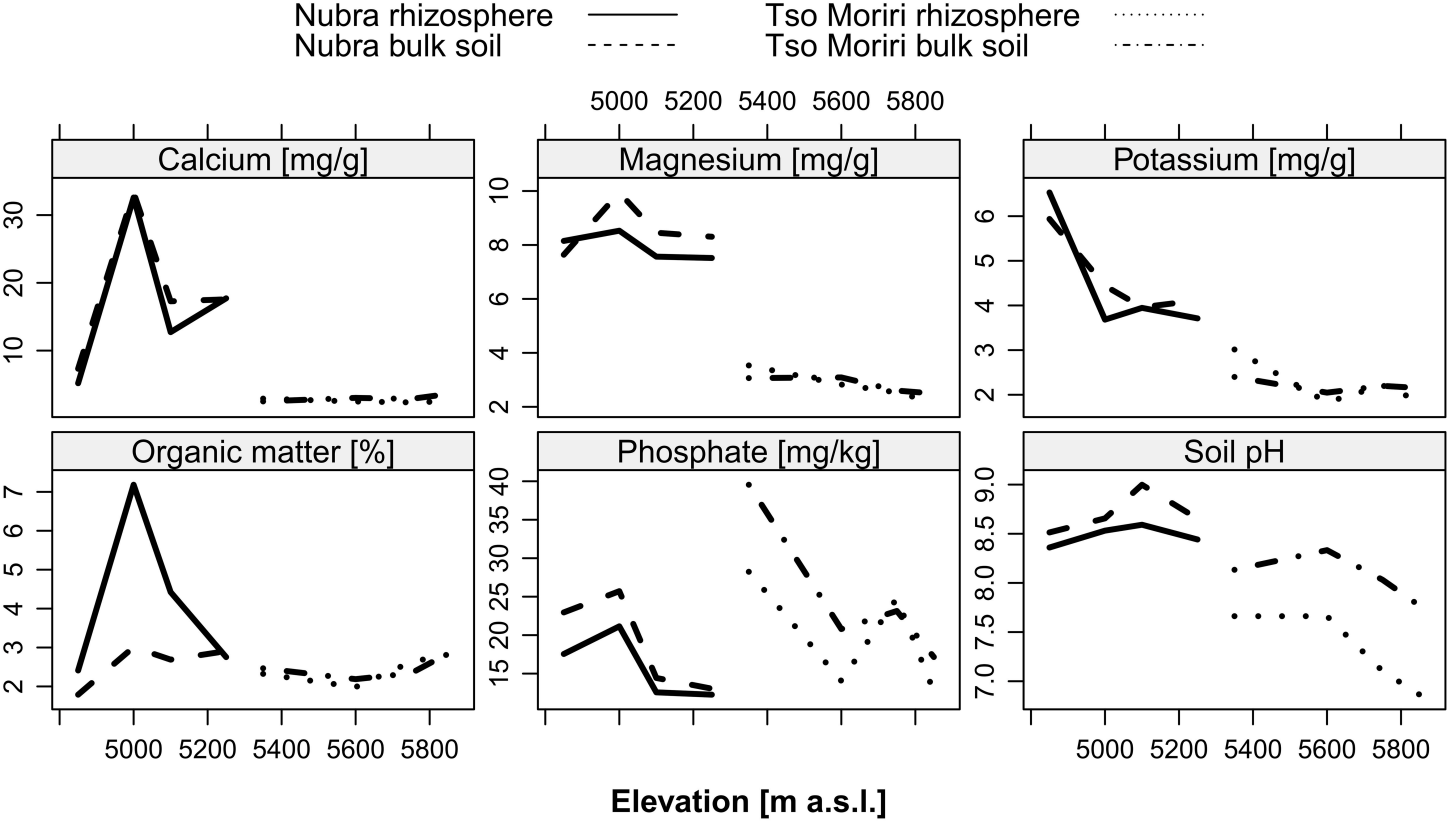
**Physico-chemical properties (Ca^2+^, Mg^+^, K+, organic matter^−^_3_, PO^3−^_4_, pH) of bulk and rhizosphere soil at two localities Nubra and Tso Moriri in all studied elevations**.

At both localities, the contents of PO^3−^_4_-P were higher in the bulk soil (Table 1, Figure 10). PO_4_-P and K content significantly decreased with elevation (Figure 10, Table 1). Sodium content increased with elevation in Tso Moriri (Table 1). Organic matter content was higher in the rhizosphere in Nubra, while in Tso Moriri the values between rhizosphere and bulk soil did not differ (Figure 10, Table 1). More Mg was found in the bulk soil at Nubra and this was significant at 5000 and 5100 m elevations. Similar magnesium concentrations were recorded in Tso Moriri with a significant decrease with elevation (Figure 10, Table 1). Calcium concentration had similar values in the rhizosphere and the bulk soil at both localities and decreased with elevation (Figure 10, Table 1). The pH had higher values in bulk soils than in the rhizosphere and the decreasing trend was observed with increasing elevation, significant in Tso Moriri (Figure 10, Table 1).

## Discussion

Vascular plants and soil microbial assemblages are indirectly connected through the soil substrate and interaction, which influence the soil fertility (Bardgett and Walker, 2004; van der Heijden et al., 2008). Higher plants contribute to resources through litter fall and rhizodeposition and can also provide suitable microclimatic conditions to belowground organisms. Microbial assemblages are able to convert the inaccessible minerals to accessible one for plants (Clarholm, 1985; Bonkowski, 2004; Carlson et al., 2010). We expected the dominant cushion plants of the high-elevation Himalaya's cold deserts to ameliorate the environmental conditions for soil microorganisms. This way, the bacterial assemblages become more diverse in the rhizosphere of *T. caespitosum* than in the bulk soil of the subnival zone. The present investigation is, to the best of our knowledge, the first study focused on the interaction between this cushion plant and its microbial assemblages in the dry Himalaya.

We found that *T. caespitosum* cushions do not have a significant impact on the total number of bacteria at both localities, and on the structure of the bacterial community in Nubra (Table 1, Figure 1). The cushions did, however, influence the bacterial community structure in Tso Moriri (Figure 2) and the cultivable part of bacterial community at both localities (Figure 4, Table 1). Hence, the cushion plants do not structure the microbial assemblages evenly along the investigated elevational gradient. The structuring effect is evident with increasing stress such as drought, nutrient and thermal limitation. The similar pattern of structuring bacterial diversity along the elevational gradient was reported from the water streams in China, where the temperature was the most important driver (Wang et al., 2012). Roy et al. (2013) found that the assemblages from the rhizosphere of *Silene acaulis* differed from the bulk soil assemblages in the French Alps. Our study indicates that such differences exist in the dry Himalayas, but they are elevation-dependent, increasing with higher abiotic stress at higher elevations. Thus, plant-bacteria association became more common as environmental conditions became less harsh and plants became more abundant (King et al., 2012).

We found a significant influence of the cushion on the cultivable part of the bacteria. The proportion of r-strategists is larger in the rhizosphere, where there is more organic matter and a lower pH, making phosphorus more accessible for organisms. The higher moisture and stable temperature also help create the conditions demanded by zymogenous (r-strategist) microorganisms (Langer et al., 2004). The rhizosphere also releases exudates (such as pentose) which are preferentially utilized by r-strategists (De Ley et al., 1993; Koch, 2001; Elliott et al., 2008; Elhottová et al., 2009). The higher proportion of K-strategists was recorded at the localities with persistent, severe conditions such as drought at lower elevations (4850–5000 m) (Cruz-Martínez et al., 2009) or regular freezing/thawing during the vegetation season at higher elevations (5750–5850 m) (Figure 9). These unfavorable conditions evoke competition about resources, which can support the growth of organisms with lower biomass turnover and high metabolic efficiency (Dworkin et al., 2006). At these localities, stable assemblages are created, capable of utilizing and exploiting the recalcitrant material. The “climax stadium” of bacterial assemblage is probably developed here.

The spatial differences in the proportion of cultivable and non-cultivable types were found along the elevational gradient and between localities (Figure 4). The relatively high degree of cultivability (1–54%) was surprising, but similar results were reported for microbial populations from other harsh environments such as sea ice (Junge et al., 2002), subglacial sediments (Foght et al., 2004) or recently from dry soil in the Andean Puna (Ferrero et al., 2010). This discovery contrasts with widely cited value (<1%) of the total count being cultivable (Amann et al., 1995). This could be caused by the relative simplicity of cold environments and their associated biota (Foght et al., 2004). The percentage of cultivable bacteria is much lower in higher elevations, especially in Tso Moriri. An increasing proportion of non-cultivable types may be associated with later successional stages since these types direct less energy into growth and/or have very specific growth requirements that may not be readily provided in artificial media (Garland et al., 2001). We suggest that generally arid soil in the southwestern part of the Tibetan Plateau, where Tso Moriri is situated, is in climax stage, not being affected by large disturbances associated with glaciation fluctuation for the millennium. Glaciological studies have indicated that contrasting patterns of glaciations exist across adjacent regions of the Himalaya, which are likely due to a combination of orographic and climatic influences (Owen et al., 2008; Dortch et al., 2010). Ice sheet glaciations did not evolve during the Last Glacial Maximum on the majority of the Tibetan Plateau (Kirchne et al., 2011). There is some evidence for the glacial advance in Korzog Range c.a. 4.7–2.7 ka BP (Leipe et al., 2014) on the other side of Tso Moriri Lake, which is way more glaciated than our study site because of northeast-facing slopes. In contrast, Nubra is a relatively young glacier forefield (up to 100 years old). The early successional states usually contain a higher proportion of cultivable types (Garland et al., 2001; Sigler et al., 2002; Krištůfek et al., 2005).

We used conditional inference trees to identify important environmental parameters structuring the bacterial assemblages in the dry Himalayan soils. A combination of locality, habitat and elevation together with soil chemical factors showed to be important predictors of variation in the bacterial assemblages. However, each parameter had different drivers. The phylogenetic structure of the bacterial community was primarily explained by the mountain range type (Tso Moriri vs. Nubra). The r-/K- strategists and numbers of cultivable bacteria were determined by the microhabitat type (inside vs. outside the cushion), while the C/T ratio was mostly influenced by elevation. Another important parameter affecting biomass and composition of bacteria was the concentration of cations, mainly sodium and calcium. Hasse et al., 2001 showed that sodium played a significant role in the energy metabolism and pathogenicity of bacteria. Also the sporulation and germination are influenced by the concentration of divalent cations, including calcium. The fast uptake of calcium was observed in stage IV of sporulation (Young and Fitz-James, 1962) and may ultimately comprise up to 3% of the dry weight of the spores (Murrell and Warth, 1965). The concentration of cations remarkably influenced the physiological properties of spores, in particular a resistance to heat, radiation, enzymes, disinfectants and other deleterious agents (Warth, 1979). We propose that the bacteria living in high-elevation soils have higher uptake of cations, which could help them to withstand severe conditions in the nival and subnival zones. This question needs to be studied more.

It is important to know which bacterial species or groups of species are present in subnival ecosystems and in the rhizosphere of the dominant vascular plant of this zone. We need to know the present species diversity, because ongoing climate warming in the Himalayas will likely influence the relative importance, frequency and composition of functional groups, their trophic interactions, and processes controlling these interactions (Chakraborty et al., 2012).

The taxonomic composition of isolated bacteria was very variable and was dominated by Actinobacteria (Table 2). Soil Actinobacteria, with a diverse machinery of enzymes, are important players in the decomposition of soil recalcitrant matter and weathering of mineral elements. Even more, mostly Streptomycetes produce phytohormones and soil enzymes, solubilize phosphate, and thus play a role as plant growth promoters (Vyas et al., 2009; Gulati et al., 2010). By contrast, Proteobacteria were a less abundant member of the studied soil assemblages. This is different from the typical soil bacterial assemblages with regular distribution of Proteobacteria, represented mainly by e.g., *Pseudomonas*, *Stenotrophomonas*, *Xanthomonas*, *Acinetobacter, Variovorax*, and other genera (Krištůfek et al., 2005; Liu et al., 2012). The increased abundance of gram-negative soil bacteria, represented mainly by α-, β-, and γ-Proteobacteria, is usually associated with rhizosphere of higher plants (Fierer et al., 2007). The *Arthrobacter* genus comprised a significant portion of the bacterial isolates from the investigated bulk soil, representing both growing strategies–fast (r-) and slow (K-), where five out of eight species were identified as r- strategists. *Streptomyces* represented relatively diversified taxa identified in both soil types (Table 2). Interestingly, six out of eight Streptomycete species (*S. badius*, *S. cirratus*, *S. cyaneofuscatus*, *S. glomeroaurantiacus*, *S. humidus*, and *S. litmocidini*) produced visible colonies early (up to day 3 of cultivation) and fitted the r-selection strategy. Other fast growing Streptomycetes, *S. rochei*, and *S. cremeus*, were described previously by Yang and Lou (2011) from spring in Carst area in China. This indicates that the *Arthrobacter* and *Streptomyces* genera cannot be assigned to the r- or K- selection category, as different species inside a particular genus differed in their ecological demands. This situation is analogous to the superior taxon Actinobacteria (Fierer et al., 2007). The community composition might be influenced by the fact that soil sample desiccation preceded the cultivation of bacteria, but the shortage of water is common for the Himalayan soils. However, there is an assumption that Actinobacteria evolved as a terrestrial clade of bacteria dominating the arid soils by an average of 64% (Battistuzzi and Hedges, 2009) and that together with Cyanobacteria they comprise the major components of Terrabacteria harboring the most developed adaptation mechanisms on terrestrial life strategy. These adaptations include desiccation resistance of the peptidoglycan layer of Gram-positive bacteria (Actinobacteria and Firmicutes), as well as spore production (Gram-positive taxa and Cyanobacteria), which confer resistance to multiple stresses (desiccation, UV radiance, or high salt concentration) typical for terrestrial habitats, and even more pronounced in mountain areas. Among the isolates, one psychrophilic strain (*Dyadobacter psychrophilus* strain Lad-5K) was detected and there are some assumptions that other strains can grow under low temperatures, being adapted to cold soils in high mountains.

This is the first study surveying the quality, quantity and life strategy of bacterial assemblages in Ladakh Mts., India along elevation gradient in association with dominant alpine plants *T. ceaspitosum*. Species composition of whole bacterial assemblages, species composition of cultivable, aerobic bacterial taxa in bulk soil and *Thylacospermum* rhizosphere were also investigated. It is apparent that physico-chemical parameters of soil are tightly coupled with the bacterial diversity in the extreme environment of mountain cold desert. The present study illuminates the complex plant-soil microbial relationship in a xeric subglacial environment and brings the primary data about diversity of heterotrophic bacteria for the Himalayan soils.

### Conflict of interest statement

The authors declare that the research was conducted in the absence of any commercial or financial relationships that could be construed as a potential conflict of interest.
